# Incidence of Diabetes Among Youth Before and During the COVID-19 Pandemic

**DOI:** 10.1001/jamanetworkopen.2023.34953

**Published:** 2023-09-21

**Authors:** Matthew T. Mefford, Rong Wei, Eva Lustigova, John P. Martin, Kristi Reynolds

**Affiliations:** 1 Department of Research and Evaluation, Kaiser Permanente Southern California, Pasadena; 2 Southern California Permanente Medical Group, Pasadena; 3 Department of Health Systems Science, Kaiser Permanente Bernard J. Tyson School of Medicine, Pasadena, California

## Abstract

**Importance:**

Prior research found increases in diabetes among youth during the COVID-19 pandemic, but few studies examined variation across sociodemographics.

**Objective:**

To examine diabetes incidence rates among a diverse population of youth in the US before and during the COVID-19 pandemic.

**Design, Setting, and Participants:**

This cohort study included data from Kaiser Permanente Southern California (KPSC) between January 1, 2016, and December 31, 2021. KPSC members aged from birth to 19 years with no history of diabetes were included. Individuals were followed up using electronic health records for diabetes incidence defined using diagnoses, laboratory values, and medications. Analyses were conducted between November 2022 and January 2023.

**Main Outcome and Measures:**

Age- and sex-standardized annual and quarterly incidence rates per 100 000 person-years (PYs) were calculated for type 1 diabetes and type 2 diabetes between 2016 and 2021. Rates were calculated within strata of age (<10 and 10-19 years), sex, and race and ethnicity (Asian/Pacific Islander, Hispanic, non-Hispanic Black, non-Hispanic White, and other/multiple/unknown). Using Poisson regression with robust error variances, incidence rate ratios (IRR) comparing 2020 to 2021 with 2016 to 2019 were calculated by diabetes type and within age, sex, and race and ethnicity strata and adjusting for health care utilization.

**Results:**

Between 2016 to 2021, there were 1200, 1100, and 63 patients with type 1 diabetes (mean [SD] age, 11.0 [4.5] years; 687 [57.3%] male), type 2 diabetes (mean [SD] age, 15.7 [2.7] years; 516 [46.9%] male), and other diabetes, respectively. Incidence of type 1 diabetes increased from 18.5 per 100 000 PYs in 2016 to 2019 to 22.4 per 100 000 PYs from 2020 to 2021 with increased IRRs among individuals aged 10 to 19 years, male individuals, and Hispanic individuals. Incidence of type 2 diabetes increased from 14.8 per 100 000 PYs from 2016 to 2019 to 24.7 per 100 000 PYs from 2020 to 2021 with increased IRRs among individuals aged 10 to 19 years, male and female individuals, and those with Black, Hispanic, and other/unknown race and ethnicity.

**Conclusions and Relevance:**

In this cohort study of youth in KPSC, incidence of diabetes increased during the COVID-19 pandemic and was more pronounced in specific racial and ethnic groups. Future research to understand differential impacts of physiologic and behavioral risk factors is warranted.

## Key Points

**Question:**

Did new-onset type 1 and type 2 diabetes increase among US youth between 2016 and 2021?

**Findings:**

In this cohort study of individuals 19 years and younger, type 1 diabetes slightly increased overall and type 2 diabetes significantly increased after the beginning of the COVID-19 pandemic, in particular among non-Hispanic Black and Hispanic youth.

**Meaning:**

These findings suggest the need for further evaluation of physiologic and behavioral risk factors preceding new-onset diabetes during the COVID-19 pandemic.

## Teaser

This cohort study examines diabetes incidence before and after the COVID-19 pandemic in individuals aged 19 years and younger.

## Introduction

Youth-onset diabetes is a serious chronic health condition, placing individuals at risk for early complications, comorbidities, and excess mortality, in particular among those who develop type 2 diabetes and those from racial and ethnic minority groups such as non-Hispanic Black individuals.^1,2^ Prior research from the SEARCH for Diabetes in Youth study showed increases in the incidence of type 1 diabetes and type 2 diabetes in the US between 2002 and 2018, with significant variability between racial and ethnic groups.^3,4^ Prior studies have reported that, in addition to traditional risk factors, respiratory viruses including influenza may influence susceptibility and trigger autoimmune responses in individuals manifesting clinical symptoms of type 1 diabetes.^5,6,7,8,9,10^ However, findings have been mixed depending on the population composition, self-report vs laboratory-confirmed infections, and duration of study follow-up for assessing diabetes risk. Further, there is less research examining the impacts of respiratory viruses on type 2 diabetes. Of more recent interest, research has shown that coronavirus-binding via angiotensin-converting enzyme 2 receptors damages islet cells and may cause acute diabetes, and hyperglycemia and insulin resistance have been noted in patients with COVID-19 infection without prior indicators of diabetes risk.^11^ Additionally, diabetes risk factors may have been exacerbated during the COVID-19 pandemic including limited physical activity, increased sedentary behaviors, sleep disturbances, and increased intake of processed foods.^12,13^

Recent studies have shown increased rates of newly diagnosed type 1 diabetes^14,15,16,17^ and type 2 diabetes^17,18,19^ in children and young adults during the COVID-19 pandemic compared with time periods prior with higher levels of glucose, hemoglobin A1_c_ (HbA_1c_), and more severe diabetic ketoacidosis. For example, a study examining pediatric diabetes between April 1, 2020, and March 31, 2021, compared with the prior 2 years found a 48% and 231% increase in the number of patients with type 1 diabetes and type 2 diabetes, respectively.^17^ However, these prior studies have been limited in examining variation by age, sex, or race and ethnicity or have been limited to shorter time periods. For the current study, we aimed to describe the incidence of new-onset type 1 diabetes and type 2 diabetes among individuals younger than 20 years in Kaiser Permanente Southern California (KPSC) between 2016 and 2021.

## Methods

### Setting

KPSC is an integrated health care delivery system with approximately 4.8 million members within a service area comprising more than 20% of Southern California’s population. KPSC membership is diverse and widely representative of the Southern California region. Members’ receipt of outpatient, inpatient, laboratory, and pharmacy services are tracked in the electronic health record system.^20^ Services performed outside of KPSC are tracked through submitted billing claims. The institutional review board at KPSC reviewed and approved the current study, and a waiver of informed consent was granted due to the data-only nature of the study. This study followed Strengthening the Reporting of Observational Studies in Epidemiology (STROBE) reporting guideline.

### Study Population

In this retrospective cohort study, we identified KPSC members younger than 20 years on December 31 of each year between 2016 and 2021 to create annual cohorts for determining diabetes incidence (eFigure 1 in Supplement 1). With the exception of individuals aged from birth to 1 year, individuals were excluded if they had less than 1 day of enrollment in the health plan the 12 months prior to their incidence year or if they had evidence of diabetes at any time prior to the incidence year. Evidence of diabetes prior to the incidence year was defined as (1) 2 or more diabetes diagnosis codes^21^ (*International Statistical Classification of Diseases and Related Health Problems, Tenth Revision *codes E08.xx, E09.xx, E10.xxx, E11.xxx, or E13.xxx, P70.2, O24.0, O24.1, O24.3, and O24.8) in any setting or (2) a patient with diabetes identified anytime previously in the SEARCH for Diabetes in Youth study. The SEARCH study criteria for defining patients with diabetes has been described elsewhere and included manual medical record reviews to confirm a diagnosis of diabetes.^22^ The total population of eligible individuals in each year ranged from 979 710 in 2016 to 1 028 997 in 2021.

### Diabetes Incidence

Patients with incident diabetes were identified using previously described methods.^23,24^ Patients with incident diabetes were defined as those meeting any of 4 criteria within a 12-month period: (1) 2 or more outpatient diagnoses of diabetes on separate days in any position, (2) 1 or more outpatient diabetes diagnoses and 1 or more outpatient prescriptions for a diabetes medication, (3) 1 or more outpatient diabetes diagnosis and 2 or more outpatient abnormal laboratory results indicative of diabetes (plasma fasting glucose ≥126 mg/dL [to convert to millimoles per liter, multiply by 0.0555], random glucose ≥200 mg/dL or HbA_1c_ ≥6.5% [to convert to proportion of total hemoglobin, multiply by 0.01]), and (4) 1 or more inpatient primary discharge diagnoses for diabetes and 1 or more outpatient indicators of diabetes (eg, diagnosis, prescription, or abnormal laboratory result). For the third criteria, if 2 of the same laboratory indicators were used, they were required to be on separate days. The date of diabetes incidence was defined as the date of the first diabetes diagnosis code.

### Diabetes Type

Next, we defined diabetes type in the 12 months following the incidence date based on the total number of diabetes diagnosis codes occurring in any inpatient, ambulatory, or virtual setting. Individuals were categorized as having type 1 diabetes if more than 50% of their diabetes diagnosis codes were E10.xx or type 2 diabetes if more than 50% of their diabetes diagnosis codes were E11.xx.^25^ Individuals who had type 1 diabetes and type 2 diabetes codes both equaling 50%, who had more than 50% of their diabetes diagnosis codes as neither type 1 diabetes nor type 2 diabetes (E08.xx, E09.xx, E13.xx, P70.2, O24.0, O24.1, O24.3, and O24.8), or who had codes where no proportion equaled 50% were categorized as having other diabetes.

### Covariates

Study characteristics of interest were defined at the diabetes diagnosis date or within the 12 months prior and included age (<10 years, 10-19 years), sex, and race and ethnicity (non-Hispanic Asian/Pacific Islander [hereafter Asian/Pacific Islander], non-Hispanic Black [hereafter Black], Hispanic, non-Hispanic White [hereafter White], and other [including Native American/Alaskan, multiracial individuals, and other race and ethnicities not listed]/unknown), body mass index, laboratory results (HbA_1c_ and fasting and random blood glucose levels), and health care utilization, defined as counts of inpatient, outpatient, emergency department, and telehealth visits.

### Statistical Analysis

Patient characteristics of type 1 diabetes and type 2 diabetes were calculated as means and SDs for continuous variables and frequencies for categorical variables overall, and for each year from 2016 to 2021. Given the small sample size of patients with other diabetes (≤5 patients in some years), characteristics for this group are not shown. []Using linear regression, trends in baseline variables between 2016 and 2021 were calculated using Cochran-Armitage and Mantel-Haenszel tests for continuous and categorical variables, respectively.

Rates of incident type 1 diabetes were defined as the number of patients with incident diabetes divided by person-time at risk and reflected as rates per 100 000 person-years (PYs) and were age- and sex-standardized to the 2010 US Census population. These rates were calculated for each year from 2016 to 2021 and, separately, by quarters 1 to 4 of each year from 2016 to 2021. For each year and, separately, quarter per year, we further calculated rates by age group (<10 years, 10-19 years), sex, and race and ethnicity. Individuals with diabetes incidence in a prior year were removed from the at-risk population for subsequent years. Next, using Poisson regression with robust error variance, we calculated incidence rate differences (IRDs) and 95% CIs comparing the combined rates of 2020 to 2021 with 2016 to 2019 overall and by age group, sex, and race and ethnicity, adjusting for health care utilization. Finally, we calculated incidence rate ratios (IRRs) and 95% CIs to examine relative differences in type 1 diabetes incidence during 2020 to 2021 compared with 2016 to 2019 as described previously. Analyses were repeated for type 2 diabetes incidence and, separately, other diabetes. To compare our results with an outcome not expected to be influenced by the pandemic, in a separate analysis we examined rates of acute appendicitis (definition in eTable 1 in Supplement 1) comparing rates in 2020 to 2021 with 2016 to 2019. All *P* values were 2-sided and *P* < .05 was considered statistically significant. Analyses were performed using SAS version 9.4 (SAS Institute). Analyses were conducted between November 2022 and January 2023.

## Results

Between 2016 and 2021, there were 1200, 1100, and 63 patients with type 1 diabetes, type 2 diabetes, and other diabetes, respectively. Among youth with incident type 1 diabetes, the mean (SD) age was 11.0 (4.5) years, with 752 individuals (62.7%) aged 10 to 19 years; the group included 628 male individuals (52.3%), and 60 Asian/Pacific Islander individuals (5.0%), 142 Black individuals (11.8%), 516 Hispanic individuals (43.0%), and 437 White individuals (36.4%) (Table 1). Among youth with type 2 diabetes, the mean (SD) age was 15.7 (2.7) years, with 1082 individuals (98.4%) aged 10 to 19 years; the group included 516 male individuals (46.9%), and 94 Asian/Pacific Islander individuals (8.5%), 151 Black individuals (13.7%), 726 Hispanic individuals (66.0%), and 83 White individuals (7.5%) (Table 1). Sociodemographics and, additionally, mean body mass index, fasting and random glucose, and HbA_1c_ levels were not different from 2016 to 2021 (*P *for trend > .05). Mean utilization increased slightly from 2016 to 2021.

**Table 1.** Characteristics of Incident Type 1 and Type 2 Diabetes, 2016-2021

Characteristic	Patients, No. (%)							*P* value trend
	Total	2016	2017	2018	2019	2020	2021	
**Type 1 diabetes**								
Observations, No.	1200	193	182	181	185	208	251	NA
Age category, y								
0-9	448 (37.3)	73 (37.8)	62 (34.1)	72 (39.8)	70 (37.8)	75 (36.1)	96 (38.2)	.82
10-19	752 (62.7)	120 (62.2)	120 (65.9)	109 (60.2)	115 (62.2)	133 (63.9)	155 (61.8)	
Sex								
Female	572 (47.7)	104 (53.9)	84 (46.2)	87 (48.1)	79 (42.7)	107 (51.4)	111 (44.2)	.18
Male	628 (52.3)	89 (46.1)	98 (53.8)	94 (51.9)	106 (57.3)	101 (48.6)	140 (55.8)	
Race and ethnicity								
Asian and Pacific Islander, Non-Hispanic	60 (5.0)	9 (4.7)	10 (5.5)	9 (5.0)	4 (2.2)	11 (5.3)	17 (6.8)	.57
Black, Non-Hispanic	142 (11.8)	27 (14.0)	23 (12.6)	23 (12.7)	14 (7.6)	29 (13.9)	26 (10.4)	
Hispanic	516 (43.0)	81 (42.0)	77 (42.3)	78 (43.1)	83 (44.9)	85 (40.9)	112 (44.6)	
White, Non-Hispanic	437 (36.4)	70 (36.3)	67 (36.8)	62 (34.3)	79 (42.7)	77 (37.0)	82 (32.7)	
Other^a^	45 (3.8)	6 (3.1)	5 (2.7)	9 (5.0)	5 (2.7)	6 (2.9)	14 (5.6)	
BMI, mean (SD)	20.1 (5.5)	19.8 (5.4)	19.7 (5.1)	20.7 (5.4)	19.6 (6.1)	21.0 (5.6)	19.8 (5.2)	.80
Fasting glucose, mean, (SD), mg/dL	238.7 (137.6)	213.6 (169.1)	220.5 (104.6)	213.5 (110.4)	233.3 (83.9)	252.8 (103.6)	291.1 (187.7)	.23
Random glucose, mean (SD), mg/dL	445.4 (196.8)	424.9 (195.8)	454.9 (208.1)	457.1 (199.5)	450.1 (188.6)	470.9 (190.0)	422.7 (199.9)	.34
HbA_1c_, mean (SD), %	11.5 (2.9)	11.0 (2.6)	11.6 (2.6)	11.6 (2.9)	11.9 (3.1)	11.5 (3.0)	11.6 (3.0)	.50
Total health care utilization, mean (SD)	13.7 (14.4)	12.9 (15.6)	12.1 (14.1)	14.6 (16.5)	13.7 (13.6)	13.1 (10.5)	15.1 (15.5)	<.001
**Type 2 diabetes**								
Observations, No.	1100	162	166	127	139	191	315	NA
Age category, y								
0-9	18 (1.6)	2 (1.2)	5 (3.0)	3 (2.4)	3 (2.2)	1 (0.5)	4 (1.3)	.30
10-19	1082 (98.4)	160 (98.8)	161 (97.0)	124 (97.6)	136 (97.8)	190 (99.5)	311 (98.7)	
Sex								
Female	584 (53.1)	97 (59.9)	89 (53.6)	68 (53.5)	76 (54.7)	93 (48.7)	161 (51.1)	.06
Male	516 (46.9)	65 (40.1)	77 (46.4)	59 (46.5)	63 (45.3)	98 (51.3)	154 (48.9)	
Race and ethnicity								
Asian and Pacific Islander, Non-Hispanic	94 (8.5)	16 (9.9)	13 (7.8)	13 (10.2)	11 (7.9)	14 (7.3)	27 (8.6)	.40
Black, Non-Hispanic	151 (13.7)	20 (12.3)	25 (15.1)	11 (8.7)	21 (15.1)	22 (11.5)	52 (16.5)	
Hispanic	726 (66.0)	112 (69.1)	110 (66.3)	87 (68.5)	90 (64.7)	130 (68.1)	197 (62.5)	
White, Non-Hispanic	83 (7.5)	11 (6.8)	15 (9.0)	10 (7.9)	14 (10.1)	12 (6.3)	21 (6.7)	
Other^a^	46 (4.2)	3 (1.9)	3 (1.8)	6 (4.7)	3 (2.2)	13 (6.8)	18 (5.7)	
BMI, mean (SD)	36.9 (8.7)	36.8 (8.9)	37.1 (9.2)	37.5 (8.1)	37.5 (10.2)	37.1 (7.2)	36.2 (8.6)	.95
Fasting glucose, mean (SD), mg/dL	160.4 (75.9)	154.5 (72.6)	140.1 (54.9)	156.7 (69.5)	150.0 (77.5)	177.0 (81.7)	168.3 (82.0)	.84
Random glucose, mean (SD), mg/dL	231.0 (142.6)	221.5 (140.1)	222.9 (172.6)	242.2 (134.7)	225.2 (138.6)	231.3 (139.6)	236.4 (135.0)	.84
HbA_1c_, mean (SD), %	8.7 (2.4)	8.4 (2.2)	8.4 (2.3)	8.7 (2.5)	8.3 (2.2)	8.8 (2.6)	9.0 (2.5)	.88
Total health care utilization, mean (SD)	12.8 (14.3)	11.1 (13.9)	12.0 (13.9)	11.7 (12.3)	13.8 (17.6)	12.1 (9.2)	14.6 (16.2)	<.001

Abbreviations: BMI, body mass index (calculated as weight in kilograms divided by height in meters squared); HbA_1c, _hemoglobin A1_c_; NA, not applicable.

SI conversion factors: To convert glucose to millimoles per liter, multiply by 0.0555; HbA_1c_ to proportion of total hemoglobin, multiply by 0.01.

^a^ Other race and ethnicity includes Native American/Alaskan, multiracial, and all other types of responses not reported in the table.

Age- and sex-standardized incidence rates of type 1 diabetes increased from 19.55 (95% CI, 16.79-22.31) per 100 000 PYs in 2016 to 24.27 (95% CI, 21.27-27.28) per 100 000 PYs in 2021 (eTable 2 in Supplement 1). Similarly, age- and sex-standardized incidence rates for type 2 diabetes increased from 15.66 (95% CI, 13.25-18.08) per 100 000 PYs in 2016 to 29.44 (95% CI, 26.19-32.69) per 100 000 PYs in 2021. Rates of other diabetes also increased from 0.98 (95% CI, 0.37-1.58) per 100 000 PYs in 2016 to 1.22 (95% CI, 0.55-1.88) per 100 000 PYs in 2021; however, case counts for this type of diabetes were small. The events, person-time, and rates for race and ethnicity groups for type 1 diabetes and type 2 diabetes (data not shown for other diabetes) are presented in eTable 3 in Supplement 1. Comparing 2020 to 2021 with 2016 to 2019, the incidence rate of type 1 diabetes was 17% higher (IRR, 1.17; 95% CI, 1.04-1.31). Type 1 diabetes incidence rates in 2020 to 2021 were also higher compared with 2016 to 2019 among those aged 10 to 19 years (IRR, 1.17; 95% CI, 1.01-1.36), male individuals (IRR, 1.18; 95% CI, 1.00-1.39), and Hispanic individuals (IRR, 1.21; 95% CI, 1.01-1.44) (Table 2). The incidence rate of type 2 diabetes was 62% higher (IRR, 1.62; 95% CI, 1.43-1.82) comparing 2020 to 2021 with 2016 to 2019. Type 2 diabetes incidence rates in 2020 to 2021 compared with 2016 to 2019 were also higher among those aged 10 to 19 years (IRR, 1.63; 95% CI, 1.44-1.84), female (IRR, 1.44; 95% CI, 1.22-1.69) and male individuals (IRR, 1.83; 95% CI, 1.54-2.17), Black individuals (IRR, 1.95; 95% CI, 1.41-2.68), Hispanic individuals (IRR, 1.61; 95% CI, 1.39-1.86), and other/unknown racial and ethnic groups (IRR, 2.96; 95% CI, 1.60-5.49). IRD followed similar patterns for type 1 diabetes and type 2 diabetes. IRR and IRD for other diabetes were not statistically significant.

**Table 2.** Adjusted IRRs and IRDs Comparing Diabetes Incidence in 2020-2021 to 2016-2019, by Diabetes Type, Age, Sex, and Race and Ethnicity

Characteristic	Rate per 100 000 PY		IRR (95% CI)	IRD (95% CI)
	2016-2019	2020-2021		
**Type 1 diabetes**				
Overall	18.09	21.13	1.17 (1.04 to 1.31)	3.03 (0.67 to 5.40)
Age group, y				
0-9	14.67	17.01	1.16 (0.96 to 1.40)	2.34 (−0.78 to 5.47)
10-19	20.93	24.47	1.17 (1.01 to 1.36)	3.54 (0.08 to 7.00)
Sex				
Female	17.67	20.29	1.15 (0.97 to 1.36)	2.61 (−0.70 to 5.93)
Male	18.51	21.84	1.18 (1.00 to 1.39)	3.33 (−0.04 to 6.69)
Race and ethnicity				
Asian and Pacific Islander, Non-Hispanic	8.00	12.82	1.60 (0.96 to 2.66)	4.82 (−0.75 to 10.39)
Black, Non-Hispanic	27.18	34.90	1.28 (0.91 to 1.80)	7.72 (−3.27 to 18.71)
Hispanic	15.39	18.55	1.21 (1.01 to 1.44)	3.17 (0.02 to 6.31)
White, Non-Hispanic	27.31	29.78	1.09 (0.90 to 1.33)	2.47 (−3.25 to 8.19)
Other^a^	8.83	9.93	1.13 (0.62 to 2.03)	1.10 (−4.53 to 6.74)
**Type 2 diabetes**				
Overall	14.56	23.53	1.62 (1.43 to 1.82)	8.96 (6.57 to 11.36)
Age group, y				
0-9	0.69	0.49	0.72 (0.26 to 2.03)	−0.19 (−0.77 to 0.39)
10-19	26.26	42.77	1.63 (1.44 to 1.84)	16.51 (12.12 to 20.89)
Sex				
Female	16.48	23.67	1.44 (1.22 to 1.69)	7.18 (3.71 to 10.66)
Male	12.73	23.26	1.83 (1.54 to 2.17)	10.53 (7.23 to 13.82)
Race and ethnicity				
Asian and Pacific Islander, Non-Hispanic	13.36	19.10	1.43 (0.95 to 2.15)	5.74 (−1.18 to 12.66)
Black, Non-Hispanic	24.02	46.79	1.95 (1.41 to 2.68)	22.77 (10.63 to 34.91)
Hispanic	19.31	31.08	1.61 (1.39 to 1.86)	11.77 (7.84 to 15.69)
White, Non-Hispanic	4.90	6.15	1.26 (0.81 to 1.95)	1.25 (−1.29 to 3.80)
Other^a^	5.36	15.89	2.96 (1.60 to 5.49)	10.52 (4.25 to 16.79)
**Other diabetes**				
Overall	1.10	0.88	0.80 (0.46 to 1.38)	−0.22 (−0.74 to 0.30)
Age group, y				
0-9	0.22	0.10	0.48 (0.05 to 4.33)	−0.11 (−0.41 to 0.18)
10-19	1.85	1.55	0.84 (0.47 to 1.47)	−0.30 (−1.24 to 0.63)
Sex				
Female	1.38	0.70	0.51 (0.22 to 1.16)	−0.68 (−1.41 to 0.05)
Male	0.83	1.05	1.26 (0.59 to 2.70)	0.22 (−0.52 to 0.95)
Race and ethnicity				
Asian and Pacific Islander, Non-Hispanic	0.51	1.44	2.82 (0.47 to 16.86)	0.93 (−0.85 to 2.70)
Black, Non-Hispanic	1.27	0	NA	−1.27 (−2.52 to −0.03)
Hispanic	1.48	1.00	0.68 (0.33 to 1.38)	−0.48 (−1.29 to 0.34)
White, Non-Hispanic	0.81	1.00	1.24 (0.40 to 3.78)	0.19 (−0.85 to 1.23)
Other^a^	0	0	NA	NA

Abbreviations: IRD, incidence rate difference; IRR, incidence rate ratio; NA, not applicable; PY, person-year.

^a^ Other race and ethnicity includes Native American/Alaskan, multiracial, and all other types of responses not reported in the table.

Quarterly rates of incident type 1 and type 2 diabetes by age group are presented in the Figure and eTable 4 in Supplement 1. Overall, rates of type 1 diabetes decreased from 26.2 per 100 000 PYs in quarter 1 (Q1) of 2016 to 18.3 per 100 000 PYs in Q4 of 2021. However, over the same period, increases in rates of incident type 1 diabetes occurred among those aged 0 to 9 years (19.7 to 23.5 per 100 000 PY). Overall, rates of incident type 2 diabetes increased from 18.4 per 100 000 PYs in Q1 of 2016 to 20.3 per 100 000 PYs in Q4 of 2021. Increases were observed over the same period among those from birth to age 9 years (0.0 to 1.7 per 100 000 PY) and aged 10 to 19 years (33.9 to 36.4 per 100 000 PY).

**Figure.**
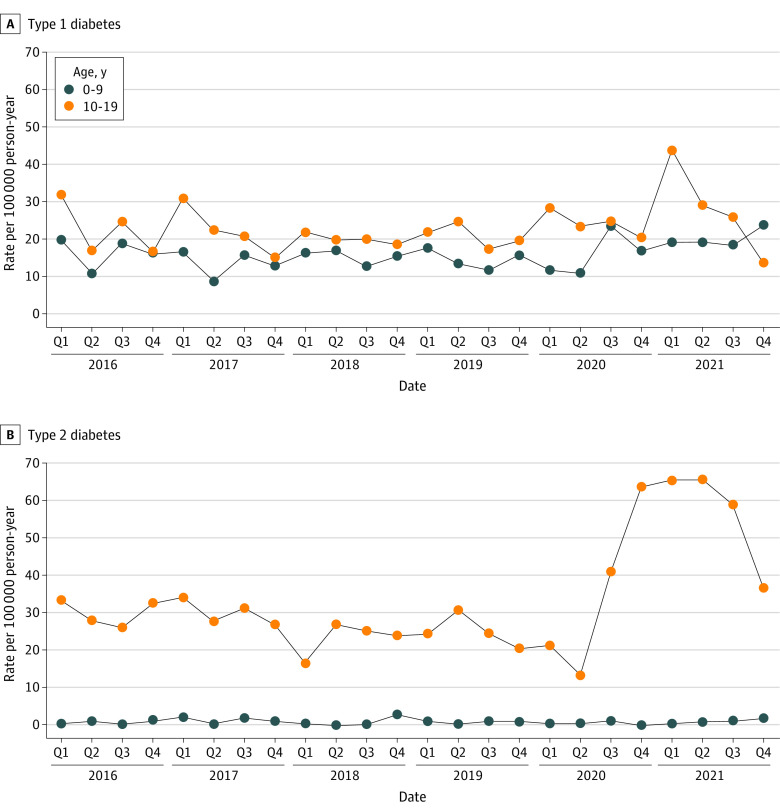
Quarterly Incidence Rates of Type 1 and Type 2 Diabetes by Age, 2016-2021 Age- and sex-standardized quarterly incidence rates for type 1 diabetes (A) and type 2 diabetes (B). Overall, incidence rates for type 1 diabetes fluctuated and slightly increased within age groups over the study period. Incidence rates for type 2 diabetes fluctuated and increased among older youth during quarter (Q) 2 of 2020.

Quarterly rates of incident type 1 diabetes and incident type 2 diabetes by sex and race and ethnicity are presented in eFigures 2 and 3 in Supplement 1, respectively, and in eTable 4 in Supplement 1. For type 1 diabetes, quarterly rates fluctuated but increased for Asian/Pacific Islander individuals (12.9 to 15.3 per 100 000 PYs). For type 2 diabetes, quarterly rates increased among male individuals (12.8 to 20.6 per 100 000 PYs) and those who were Asian/Pacific Islander (17.2 to 22.9 per 100 000 PYs), Black (30.1 to 43.2 per 100 000 PYs), and White (3.3 to 9.6 per 100 000 PYs). Rates of incident type 2 diabetes had large increases among both sexes and Black and Hispanic individuals between Q2 and Q4 of 2020. Similar increases in magnitude were not observed for type 1 diabetes. Separately, rates of acute appendicitis fluctuated between 2016 and 2021 but were not higher from 2020 to 2021 compared with 2016 to 2019 (IRR, 0.92; 95% CI, 0.88-0.97) (eTables 5-6 in Supplement 1).

## Discussion

Incidence rates of both type 1 diabetes and type 2 diabetes increased during the COVID-19 pandemic compared with before. Increases were observed overall and within specific strata of age, in particular, those aged 10 to 19 years, male individuals, and among some racial and ethnic groups. By quarter, rates of type 1 diabetes fluctuated seasonally but increased among those aged 10 to 19 years during Q1 of 2021. Among individuals with type 2 diabetes, a spike in incidence was observed during Q3 and Q4 of 2020, especially among Black and Hispanic individuals, suggesting a disproportionate burden of type 2 diabetes among these youth during the pandemic period of our study.

Consistent with our findings, prior studies reported increases type 1 and type 2 diabetes incidence among youth in the years before the COVID-19 pandemic. The SEARCH study reported that incidence of type 1 and type 2 diabetes increased to 22.2 per 100 000 PYs and 17.9 per 100 000 PYs, respectively, by 2017 to 2018 with rates of increase generally higher among racial and ethnic minority populations.^3^ Our findings also align with studies that noted increases in incidence of type 1 and type 2 diabetes in youth during the COVID-19 pandemic across the US.^15,16,17,26^ A study of 3113 individuals younger than 21 years from 24 clinical sites found that the number of individuals with type 2 diabetes increased by 77% in 2020 compared with 2018 and 2019. While many of these studies either examined proportional increases in cases, case counts, or reported diabetes complications among new cases during the pandemic, few calculated incidence rates. There are several potential mechanisms by which the pandemic has impacted rates of incident type 1 and type 2 diabetes. At a biological level, SARS-CoV-2, the virus underlying COVID-19, has been shown to damage pancreatic cells by infection or immune dysregulation. Acute stress of SARS-CoV-2 infection has been shown to increase blood glucose levels, and individuals with previously undiagnosed diabetes may receive a diagnosis when presenting to hospitals for infection-related care.^27^ Further, the increase in sedentary behavior or stressors promoted by the pandemic may have resulted in weight gain and other negative health effects increasing the risk of diabetes.^28^

The COVID-19 pandemic has been a challenge for health care systems with stay-at-home orders, suspension of nonessential hospital visits, the switch to virtual-based care limiting patient visits resulting in missed screenings and potential gaps in care. Within KPSC, while there were no initiatives for increased diabetes screening among at-risk patients, the shift to increased virtual care allowed for better electronic monitoring of patients’ health status, which may account for some additional patients with diabetes detected during the pandemic years. Among the few studies examining incidence rates during the pandemic, increases have been observed but rates have varied between studies. For example, a study of individuals with diabetes among those younger than 18 years in a multiregion, Florida-based health network found that age-standardized rates of type 1 and type 2 diabetes ranged from 19.9-32.5 per 100 000 PYs and 10.6-14.6 per 100 000 PYs, respectively, prior to the pandemic and increased to between 31.8 to 36.3 per 100 000 PYs (type 1 diabetes) and 13.1 to 16.9 per 100 000 PYs (type 2 diabetes) during the pandemic.^26^ By contrast, we observed a smaller overall increase in type 1 diabetes incidence but a more pronounced increase in type 2 diabetes incidence during the pandemic, in particular among those aged 10 to 19 years; given the small number of patients with type 2 diabetes in those aged from birth to 9 years, we interpret these results with caution. These differences may be related to population composition, ie, youth in the southern US vs southwestern US, or case definitions of incident diabetes. Additionally, changes in the underlying denominator populations or changes in care-seeking behavior related to the pandemic may explain some variation in incidence during our study period. However, after adjusting for use prior to diabetes incidence, our results were minimally attenuated. In our study, baseline clinical characteristics of patients with incident type 1 diabetes and type 2 diabetes were not different during nonpandemic and pandemic years. This is consistent with other studies showing similarity in age, HbA_1c_, and body mass index among patients with incident diabetes before and during the pandemic.^15^

### Strengths and Limitations

Strengths of the current study include a large, diverse population of children and youth representative of the southern California region, with comprehensive health records and near complete capture of clinically relevant covariates. We also acknowledge some limitations. Our study population is limited to members with health insurance and may not be fully generalizable to a less-insured population. However, we age- and sex-standardized rates to the US Census population for comparability. COVID-19 incidence at infection was not assessed; therefore, we cannot make connections to underlying pathophysiologic or inflammatory mechanisms that may promote the development of diabetes. Medical record review was not conducted to confirm diabetes type; however, we used a prior validated method that considers the proportions of diabetes diagnosis codes with high sensitivity and specificity. Sociocultural and behavioral factors that may impact the risk in diabetes incidence before and during the COVID-19 pandemic periods were not examined in the current study and we are unable to account for the potential differential impact of these factors on our subgroups of interest. Although we adjusted for care-seeking behavior using health care utilization, it is possible that unmeasured confounding may influence our associations. Further, we did not test for statistical differences in IRD or IRR between subgroups of age, sex, or race and ethnicity. Finally, we did not examine the severity or presentation of diabetic ketoacidosis at diabetes incidence, which may be considered as a future direction.

## Conclusions

The incidence of both type 1 and type 2 diabetes among youth increased following the start of the COVID-19 pandemic with the largest increases observed among Black and Hispanic individuals. Future research to understand potential underlying physiologic and behavioral risk factors before and during the pandemic among individuals at high risk for incident diabetes is warranted.

## Notes

Supplement 1. **eFigure 1.** Cohort flow chart **eFigure 2. **Quarterly incidence rates of type 1 diabetes by sex, and race/ethnicity, 2016-2021 **eFigure 3.** Quarterly incidence rates of type 2 diabetes by sex, and race/ethnicity, 2016-2021 **eTable 1.** Definition for acute appendicitis **eTable 2.** Events, person-time and rates of incident diabetes by diabetes type, 2016-2021 **eTable 3.** Events, person-time, and rates of incident diabetes by diabetes type and race/ethnicity, 2016-2021 **eTable 4.** Quarterly incidence rates per 100,000 person-years for incident type 1 and 2 diabetes overall and by age, sex, and race/ethnicity, 2016-2021 **eTable 5.** Quarterly incidence rates per 100,000 person-years for acute appendicitis overall and by age, sex, and race/ethnicity, 2016-2021 **eTable 6.** Incidence rate ratios comparing acute appendicitis in 2020-2021 to 2016-2019, by diabetes type, age, sex, and race/ethnicity

Supplement 2.
Data Sharing Statement
