# Supplementary material for: Incidence of Diabetes Among Youth Before and During the COVID-19 Pandemic
Source: JAMA Netw Open. 2023 Sep 21;6(9):e2334953. doi: 10.1001/jamanetworkopen.2023.34953 (PMC10514735; doi:10.1001/jamanetworkopen.2023.34953)
Supplement: Supplement 1. — eFigure 1. Cohort flow chart eFigure 2. Quarterly incidence rates of type 1 diabetes by sex, and race/ethnicity, 2016-2021 eFigure 3. Quarterly incidence rates of type 2 diabetes by sex, and race/ethnicity, 2016-2021 eTable 1. Definition for acute appendicitis eTable 2. Events, person-time and rates of incident diabetes by diabetes type, 2016-2021 eTable 3. Events, person-time, and rates of incident diabetes by diabetes type and race/ethnicity, 2016-2021 eTable 4. Quarterly incidence rates per 100,000 person-years for incident type 1 and 2 diabetes overall and by age, sex, and race/ethnicity, 2016-2021 eTable 5. Quarterly incidence rates per 100,000 person-years for acute appendicitis overall and by age, sex, and race/ethnicity, 2016-2021 eTable 6. Incidence rate ratios comparing acute appendicitis in 2020-2021 to 2016-2019, by diabetes type, age, sex, and race/ethnicity [file jamanetwopen-e2334953-s001.pdf]

Supplemental Online Content

Mefford MT, Wei R, Lustigova E, Martin JP, Reynolds K. Incidence of diabetes among youth before and during the COVID-19 pandemic. *JAMA Netw Open*. 2023;6(9):e2334953. doi:10.1001/jamanetworkopen.2023.34953

eFigure 1. Cohort flow chart

eFigure 2. Quarterly incidence rates of type 1 diabetes by sex, and race/ethnicity, 2016-2021

eFigure 3. Quarterly incidence rates of type 2 diabetes by sex, and race/ethnicity, 2016-2021

eTable 1. Definition for acute appendicitis

eTable 2. Events, person-time and rates of incident diabetes by diabetes type, 2016-2021

eTable 3. Events, person-time, and rates of incident diabetes by diabetes type and race/ethnicity, 2016-2021

eTable 4. Quarterly incidence rates per 100,000 person-years for incident type 1 and 2 diabetes overall and by age, sex, and race/ethnicity, 2016-2021

eTable 5. Quarterly incidence rates per 100,000 person-years for acute appendicitis overall and by age, sex, and race/ethnicity, 2016-2021

eTable 6. Incidence rate ratios comparing acute appendicitis in 2020-2021 to 2016-2019, by diabetes type, age, sex, and race/ethnicity

This supplemental material has been provided by the authors to give readers additional information about their work.

© 2023 Mefford MT et al. *JAMA Network Open.*

eTable 1. Definition for acute appendicitis

Outcome	Definition	ICD-10 Codes
**Acute Appendicitis**	≥1 inpatient principal discharge or emergency department diagnosis	K35.2, K35.2, K35.80, K35.89

Abbreviations: ICD-10, International Classification of Diseases, 10th Revision

© 2023 Mefford MT et al. *JAMA Network Open.*

eTable 2. Events, person-time and rates of incident diabetes by diabetes type, 2016-2021

		Year					
		2016	2017	2018	2019	2020	2021
**Type 1 Diabetes**	Events	193	182	181	185	208	251
	Person-Years	977,791	996,769	1,008,854	1,022,358	1,024,928	1,026,793
	Crude rate per 100,000 PY (95% Confidence Interval)	19.74 (17.14-22.73)	18.26 (15.79-21.11)	17.94 (15.51-20.75)	18.10 (15.67-20.90)	20.29 (17.72-23.25)	24.45 (21.60-27.66)
	Standardized[a] rate per 100,000 PY (95% Confidence Interval)	19.55 (16.79-22.31)	18.02 (15.40-20.64)	17.83 (15.23-20.43)	17.97 (15.38-20.56)	20.12 (17.38-22.85)	24.27 (21.27-27.28)
**Type 2 Diabetes**	Events	162	166	127	139	191	315
	Person-Years	977,791	996,769	1,008,854	1,022,358	1,024,928	1,026,793
	Crude rate per 100,000 PY (95% Confidence Interval)	16.57 (14.20-19.33)	16.65 (14.30-19.39)	12.59 (10.58-14.98)	13.60 (11.51-16.05)	18.64 (16.17-21.47)	30.68 (27.47-34.26)
	Standardized[a] rate per 100,000 PY (95% Confidence Interval)	15.66 (13.25-18.08)	15.87 (13.45-18.28)	12.06 (9.96-14.15)	13.07 (10.90-15.24)	17.90 (15.36-20.44)	29.44 (26.19-32.69)
**Other Diabetes**	Events	10	7	13	14	5	13
	Person-Years	977,791	996,769	1,008,854	1,022,358	1,024,928	1,026,793
	Crude rate per 100,000 PY (95% Confidence Interval)	1.02 (0.55-1.90)	0.70 (0.33-1.47)	1.29 (0.75-2.22)	1.37 (0.81-2.31)	0.49 (0.20-1.17)	1.27 (0.74-2.18)
	Standardized[a] rate per 100,000 PY (95% Confidence Interval)	0.98 (0.37-1.58)	0.68 (0.18-1.18)	1.24 (0.57-1.91)	1.32 (0.63-2.02)	0.48 (0.06-0.89)	1.22 (0.55-1.88)

Abbreviations: PY, person-years
[a]Age- and sex-standardized to the 2010 US Census population

© 2023 Mefford MT et al. *JAMA Network Open.*

eTable 3. Events, person-time, and rates of incident diabetes by diabetes type and race/ethnicity, 2016-2021

**Type 1 Diabetes**							
		**Year**					
		2016	2017	2018	2019	2020	2021
Asian and Pacific Islander, Non-Hispanic	Events	9	10	9	< 5	11	17
	Person-Years	92,871	96,595	99,941	103,176	104,143	104,908
	Crude rate per 100,000 PY (95% CI)	9.69 (7.37-13.03)	10.35 (6.83-12.12)	9.01 (6.74-11.95)	3.88 (6.84-12.10)	10.56 (7.75-13.60)	16.20 (9.45-16.40)
Black, Non-Hispanic	Events	27	23	23	14	29	26
	Person-Years	79,617	79,099	78,152	77,336	75,789	74,091
	Crude rate per 100,000 PY (95% CI)	33.91 (24.54-37.23)	29.08 (22.72-34.68)	29.43 (22.37-34.20)	18.10 (22.70-34.64)	38.26 (25.75-38.89)	35.09 (31.48-46.82)
Hispanic	Events	81	77	78	83	85	112
	Person-Years	505,564	509,194	507,493	507,223	502,339	497,903
	Crude rate per 100,000 PY (95% CI)	16.02 (14.39-19.61)	15.12 (13.31-18.29)	15.37 (13.11-18.03)	16.36 (13.30-18.26)	16.92 (15.12-20.45)	22.49 (18.54-24.55)
White, Non-Hispanic	Events	70	67	62	79	77	82
	Person-Years	240,704	246,083	251,060	253,830	252,246	249572
	Crude rate per 100,000 PY (95% CI)	29.08 (24.57-33.84)	27.23 (22.74-31.53)	24.70 (22.40-31.08)	31.12 (22.75-31.46)	30.53 (25.86-35.25)	32.86 (31.69-42.32)
Other[a]	Events	6	5	9	5	6	14
	Person-Years	59,035	65,798	72,208	80,793	90,411	100,319
	Crude rate per 100,000 PY (95% CI)	10.16 (6.75-12.85)	7.60 (6.26-11.95)	12.46 (6.17-11.77)	6.19 (6.27-11.91)	6.64 (7.11-13.37)	13.96 (8.68-16.12)
**Type 2 Diabetes**							
		2016	2017	2018	2019	2020	2021
Asian and Pacific Islander, Non-Hispanic	Events	16	13	13	11	14	27
	Person-Years	92,871	96,595	99,941	103,176	104,143	104,908
	Crude rate per 100,000 PY (95% CI)	17.23 (10.85-17.82)	13.46 (11.00-18.01)	13.01 (8.27-13.92)	10.66 (9.05-15.06)	13.44 (12.67-20.45)	25.74 (21.35-33.28)
Black, Non-Hispanic	Events	20	25	11	21	22	52
	Person-Years	79,617	79,099	78,152	77,336	75,789	74,091
	Crude rate per 100,000 PY (95% CI)	25.12 (23.71-36.30)	31.61 (24.03-36.71)	14.08 (18.02-28.44)	27.15 (19.71-30.77)	29.03 (27.66-41.71)	70.18 (46.72-67.70)

© 2023 Mefford MT et al. *JAMA Network Open.*

Hispanic	Events	112	110	87	90	130	197
	Person-Years	505,564	509,194	507,493	507,223	502,339	497,903
	Crude rate per 100,000 PY (95% CI)	22.15 (18.35-25.23)	21.60 (18.60-25.50)	17.14 (13.88-19.85)	17.74 (15.21-21.44)	25.88 (21.48-28.89)	39.57 (36.63-46.44)
White, Non-Hispanic	Events	11	15	10	14	12	21
	Person-Years	240,704	246,083	251,060	253,830	252,246	249,572
	Crude rate per 100,000 PY (95% CI)	4.57 (3.85-6.45)	6.10 (3.90-6.52)	3.98 (2.93-5.03)	5.52 (3.21-5.45)	4.76 (4.49-7.41)	8.41 (7.55-12.07)
Other[a]	Events	< 5	< 5	6	< 5	13	18
	Person-Years	59,035	65,798	72,208	80,793	90,411	100,319
	Crude rate per 100,000 PY (95% CI)	5.08 (6.08-11.66)	4.56 (6.17-11.78)	8.31 (4.66-9.07)	3.71 (5.09-9.82)	14.38 (7.11-13.37)	17.94 (11.95-21.82)

[a]Other race and ethnicity includes multiracial, Native American/Alaskan, and all other types of responses not reported in the tableSmall cell counts represented as < 5

© 2023 Mefford MT et al. *JAMA Network Open.*

eTable 4. Quarterly incidence rates per 100,000 person-years for incident type 1 and 2 diabetes overall and by age, sex, and race/ethnicity, 2016-2021

	2016				2017				2018				2019				2020				2021			
	Q1	Q2	Q3	Q4	Q1	Q2	Q3	Q4	Q1	Q2	Q3	Q4	Q1	Q2	Q3	Q4	Q1	Q2	Q3	Q4	Q1	Q2	Q3	Q4
**Type 1 Diabetes**																								
**Overall**	26.2	14.3	22.1	16.4	24.5	16.1	18.5	14.0	19.4	18.6	16.7	17.0	20.0	19.6	14.9	18.0	20.7	17.6	24.2	18.7	32.3	24.5	22.6	18.3
**0-9 years**	19.7	10.7	18.8	16.1	16.6	8.7	15.7	13.1	16.3	17.1	12.8	15.4	17.7	13.5	11.8	16.0	11.7	10.9	23.5	16.8	19.3	19.3	18.4	23.5
**10-19 years**	31.6	17.3	24.9	16.6	31.2	22.3	20.8	14.9	22.2	19.9	19.9	18.5	22.0	24.9	17.6	19.8	28.5	23.4	24.8	20.5	43.7	29.1	26.2	13.8
**Female**	32.7	15.1	19.3	20.1	22.2	17.2	17.2	12.3	17.8	20.3	16.2	16.2	21.6	15.2	11.2	15.2	27.9	14.4	23.9	19.1	32.6	22.3	17.5	15.9
**Male**	20.0	13.6	24.8	12.8	26.7	14.9	19.6	15.7	21.0	17.1	17.1	17.8	18.4	23.7	18.4	20.7	13.8	20.6	24.4	18.3	32.0	26.7	27.5	20.6
**Asian and Pacific Islander, Non-Hispanic**	12.9	0.0	21.5	4.3	16.6	20.7	4.1	0.0	8.0	12.0	12.0	4.0	3.9	3.9	0.0	7.8	23.0	11.5	7.7	0.0	19.1	11.4	19.1	15.3
**Black, Non-Hispanic**	45.2	20.1	45.2	25.1	30.3	30.3	40.5	15.2	20.5	30.7	35.8	30.7	15.5	25.9	15.5	15.5	47.5	31.7	21.1	52.8	27.0	64.8	10.8	37.8
**Hispanic**	20.6	11.1	15.8	16.6	22.0	14.9	10.2	13.4	18.9	15.8	13.4	13.4	14.2	20.5	10.3	20.5	14.3	10.4	27.1	15.9	29.7	20.9	24.1	15.3
**White, Non-Hispanic**	41.5	26.6	28.3	19.9	34.1	16.3	35.8	22.8	25.5	25.5	22.3	25.5	44.1	26.8	29.9	23.6	30.1	34.9	30.1	27.0	46.5	28.8	33.7	22.4
**Other[a]**	6.8	6.8	20.3	6.8	12.2	0.0	12.2	6.1	16.6	11.1	5.5	16.6	5.0	5.0	14.9	0.0	4.4	4.4	13.3	4.4	27.9	15.9	0.0	12.0
**Type 2 Diabetes**																								
**Overall**	18.4	15.5	14.3	18.0	19.3	14.8	17.7	14.8	8.7	14.3	13.5	13.9	13.3	16.4	13.3	11.3	11.3	7.0	22.2	34.0	35.1	35.5	31.9	20.3
**0-9 years**	0.0	0.9	0.0	0.9	1.7	0.0	1.7	0.9	0.0	0.0	0.0	2.6	0.8	0.0	0.8	0.8	0.0	0.0	0.8	0.0	0.0	0.8	0.8	1.7
**10-19 years**	33.9	27.9	26.4	32.4	34.2	27.5	31.2	26.7	16.3	26.6	25.1	23.6	24.2	30.7	24.2	20.5	21.2	13.1	40.9	63.5	65.5	65.5	59.0	36.4
**Female**	24.3	20.9	15.1	20.9	18.9	14.0	22.2	18.1	9.7	13.0	13.0	19.5	16.0	19.2	14.4	11.2	9.6	8.0	23.1	33.5	33.4	35.8	39.0	19.9
**Male**	12.8	10.4	13.6	15.2	19.6	15.7	13.3	11.8	7.8	15.5	14.0	8.5	10.7	13.8	12.3	11.5	13.0	6.1	21.4	34.4	36.6	35.1	25.2	20.6
**Asian and Pacific Islander, Non-Hispanic**	17.2	8.6	17.2	25.8	20.7	16.6	12.4	4.1	4.0	20.0	4.0	24.0	7.8	7.8	23.3	3.9	11.5	7.7	15.4	19.2	19.1	34.3	26.7	22.9
**Black, Non-Hispanic**	30.1	20.1	30.1	20.1	25.3	25.3	60.7	15.2	10.2	5.1	15.4	25.6	46.6	15.5	25.9	20.7	21.1	10.6	21.1	63.3	75.6	108.0	54.0	43.2
**Hispanic**	25.3	22.9	17.4	22.9	25.9	17.3	20.4	22.8	13.4	20.5	18.1	16.6	13.4	25.2	15.8	16.6	15.1	10.4	30.3	47.8	48.2	42.6	42.6	24.9
**White, Non-Hispanic**	3.3	3.3	5.0	6.6	6.5	8.1	3.3	6.5	3.2	4.8	4.8	3.2	9.5	4.7	3.2	4.7	4.8	1.6	4.8	7.9	11.2	4.8	8.0	9.6
**Other[a]**	6.8	6.8	0.0	6.8	6.1	6.1	6.1	0.0	0.0	5.5	22.2	5.5	0.0	9.9	5.0	0.0	0.0	0.0	35.4	22.1	15.9	23.9	27.9	4.0

[a]Other race and ethnicity includes multiracial, Native American/Alaskan, and all other types of responses not reported in the table

© 2023 Mefford MT et al. *JAMA Network Open.*

eTable 5. Quarterly incidence rates per 100,000 person-years for acute appendicitis overall and by age, sex, and race/ethnicity, 2016-2021

	2016				2017				2018				2019				2020				2021			
	**Q1**	**Q2**	**Q3**	**Q4**	**Q1**	**Q2**	**Q3**	**Q4**	**Q1**	**Q2**	**Q3**	**Q4**	**Q1**	**Q2**	**Q3**	**Q4**	**Q1**	**Q2**	**Q3**	**Q4**	**Q1**	**Q2**	**Q3**	**Q4**
**Overall**	122.5	125.8	138.6	111.4	121.8	119.0	126.2	114.1	129.9	127.5	130.3	123.5	117.1	118.7	136.0	125.4	107.4	113.3	120.8	125.9	101.8	128.0	119.4	102.9
**0-9 years**	50.2	61.0	72.6	67.2	58.5	68.1	74.3	57.7	68.5	76.2	74.5	77.1	58.9	73.2	75.7	63.9	61.2	48.7	67.1	70.5	45.3	64.6	62.1	54.5
**10-19 years**	183.8	180.8	194.5	148.9	176.0	162.5	170.8	162.5	183.2	172.0	178.7	163.8	168.2	158.6	188.9	179.3	148.0	170.1	167.9	174.5	151.1	183.4	169.5	145.3
**Female**	109.2	94.1	103.4	103.4	96.4	98.0	121.9	89.8	114.7	91.9	104.9	101.7	97.1	97.9	111.6	93.1	100.9	92.9	102.5	99.3	81.5	91.1	107.1	78.3
**Male**	135.3	156.2	172.3	119.1	146.1	139.0	130.3	137.4	144.4	161.6	154.6	144.4	136.4	138.7	159.5	156.4	113.7	132.9	138.3	151.4	121.2	163.4	131.2	126.6
**Asian and Pacific Islander, Non-Hispanic**	64.7	60.4	95.0	51.8	91.3	37.3	107.9	37.3	60.2	56.1	64.2	56.1	23.3	58.3	93.2	73.8	34.6	57.7	77.0	88.5	72.6	68.8	49.7	45.9
**Black, Non-Hispanic**	70.4	35.2	75.4	35.2	55.6	70.8	75.9	75.9	35.8	66.5	61.4	61.4	87.9	41.4	62.1	46.6	21.1	52.8	31.7	47.5	54.0	48.6	32.4	27.0
**Hispanic**	161.9	173.9	185.1	161.9	151.2	148.9	167.1	146.5	186.7	170.8	170.8	156.5	159.8	155.8	190.0	164.5	154.1	153.3	161.3	173.3	138.4	171.6	166.0	144.9
**White, Non-Hispanic**	100.0	103.3	100.0	76.6	117.3	127.1	88.0	104.3	92.6	115.0	122.9	116.6	107.4	115.3	99.5	116.9	93.8	104.9	112.8	98.5	72.3	120.5	114.1	98.0
**Other[a]**	40.7	33.9	54.2	20.3	36.5	36.5	42.6	60.8	61.0	33.3	38.8	77.6	29.7	49.5	39.6	49.5	44.3	31.0	44.3	48.7	59.8	51.9	39.9	23.9

[a]Other race and ethnicity includes multiracial, Native American/Alaskan, and all other types of responses not reported in the table

© 2023 Mefford MT et al. *JAMA Network Open.*

eTable 6. Incidence rate ratios comparing acute appendicitis in 2020-2021 to 2016-2019, by diabetes type, age, sex, and race/ethnicity

	2016 - 2019	2020 - 2021	
	Rate per 100,00 PY	Rate per 100,000 PY	IRR (95% CI)
Overall	124.24	114.92	0.92 (0.88, 0.97)
Age group, years			
0-9	67.43	59.24	0.88 (0.80, 0.97)
10-19	173.27	163.71	0.94 (0.89, 1.00)
Female	101.81	94.17	0.92 (0.86, 1.00)
Male	145.76	134.85	0.93 (0.87, 0.99)
Asian and Pacific Islander, Non-Hispanic	64.32	61.85	0.96 (0.78, 1.19)
Black, Non-Hispanic	59.84	39.36	0.66 (0.49, 0.88)
Hispanic	165.69	157.89	0.95 (0.90, 1.01)
White, Non-Hispanic	106.53	101.87	0.96 (0.86, 1.06)
Other[a]	44.29	43.02	0.97 (0.73, 1.28)

[a]Other race and ethnicity includes multiracial, Native American/Alaskan, and all other types of responses not reported in the table

© 2023 Mefford MT et al. *JAMA Network Open.*

eFigure 1. Cohort flow chart

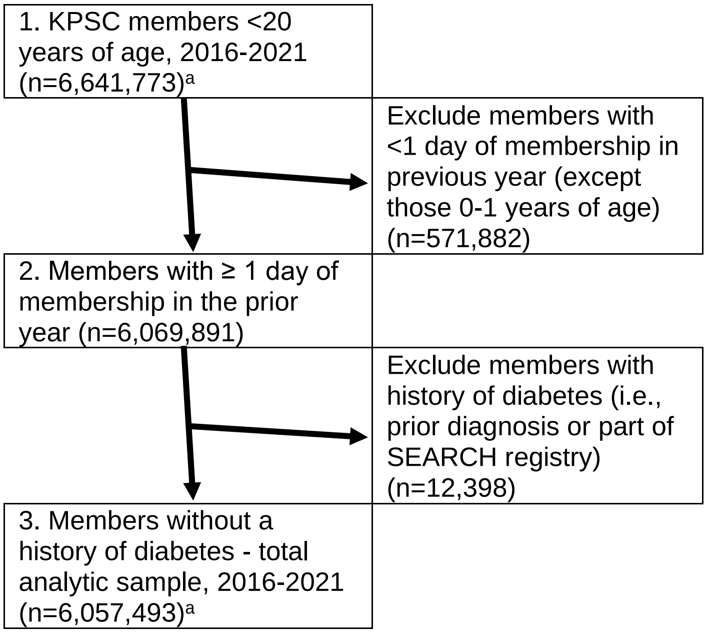

[a]Reflects non-unique member counts across all study years, where individuals without incident diabetes were eligible for subsequent years during the study period. Annual cohort sizes ranged from approximately 900,000 to 1 million youth across each year.

© 2023 Mefford MT et al. *JAMA Network Open.*

eFigure 2. Quarterly incidence rates of type 1 diabetes by sex, and race/ethnicity, 2016-2021

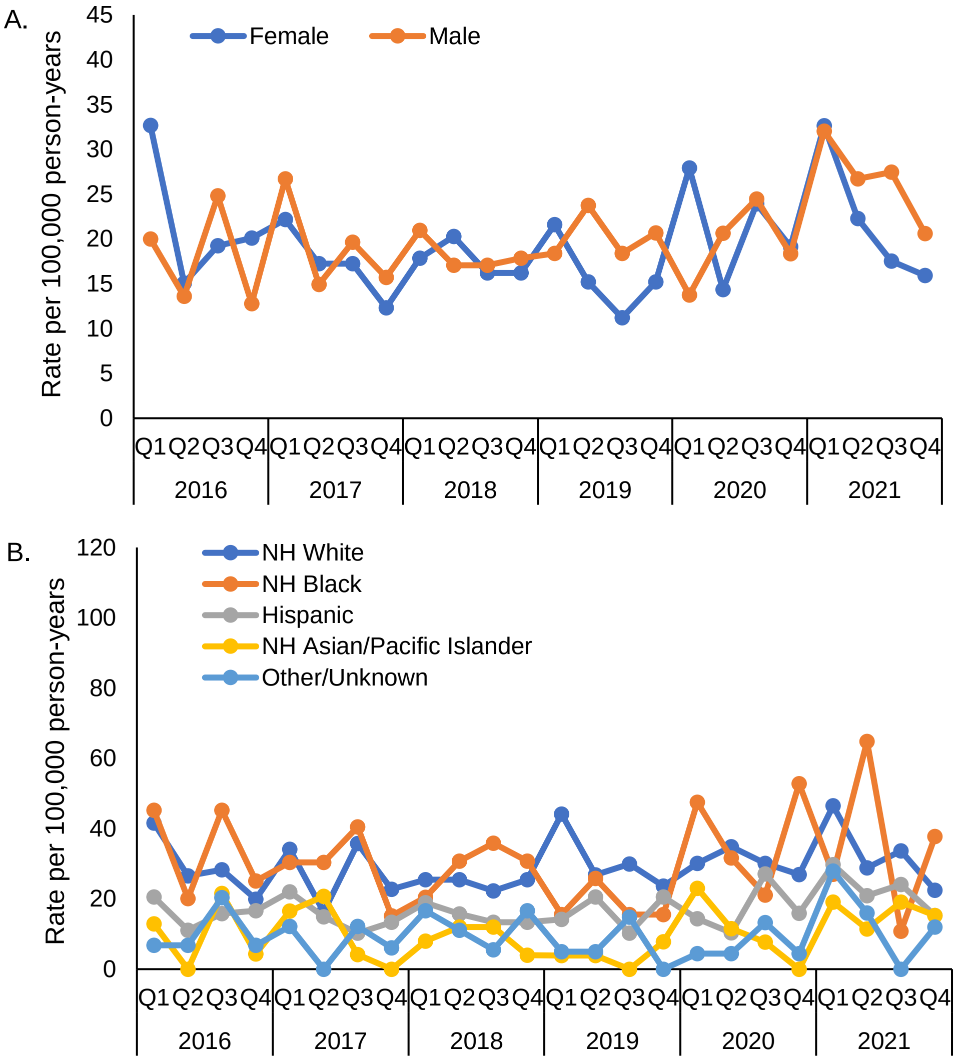

© 2023 Mefford MT et al. *JAMA Network Open.*

eFigure 3. Quarterly incidence rates of type 2 diabetes by sex, and race/ethnicity, 2016-2021

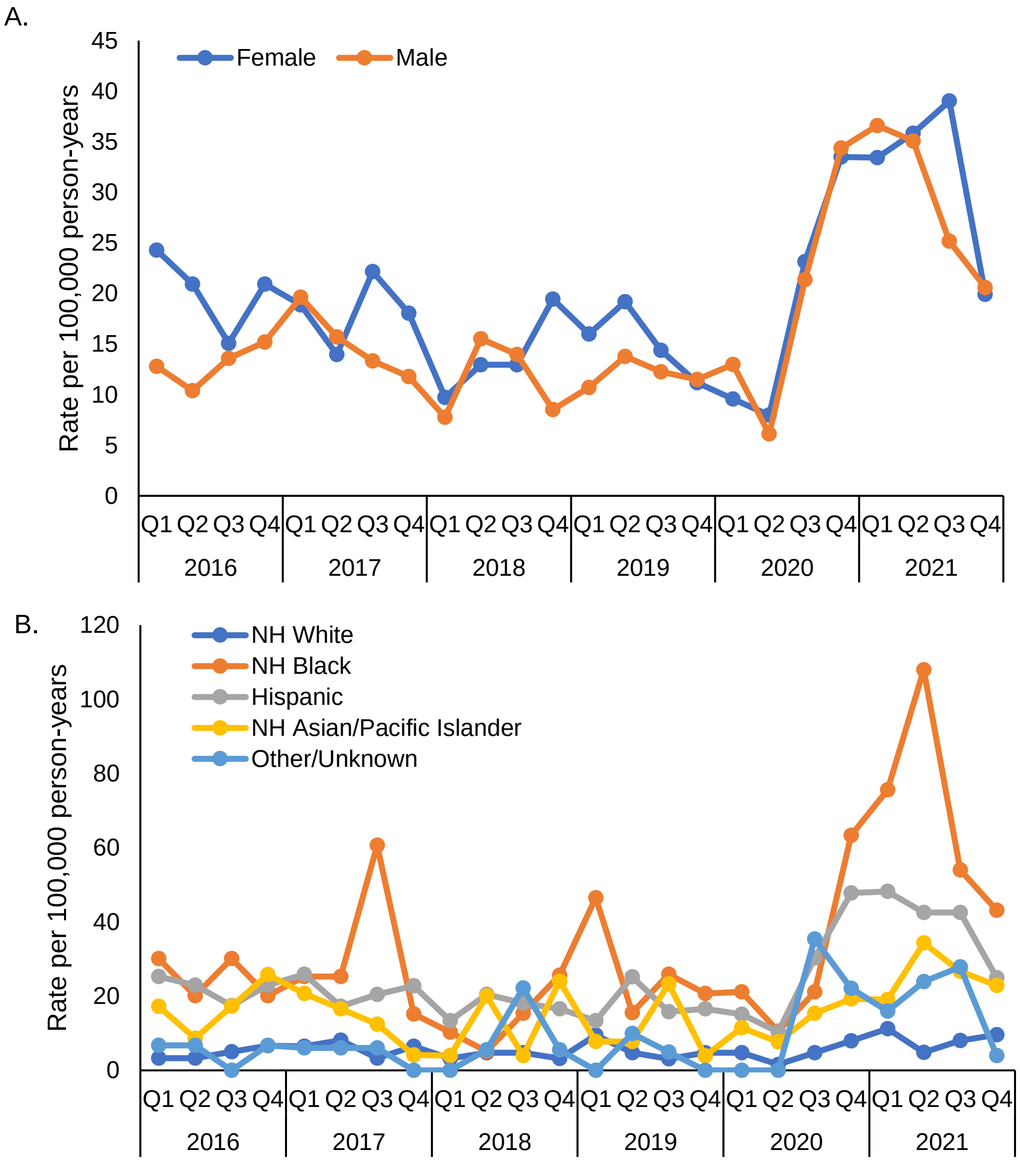

© 2023 Mefford MT et al. *JAMA Network Open.*
