# Supplementary material for: Incidence of Diabetes Among Youth Before and During the COVID-19 Pandemic
Source: JAMA Netw Open. 2023 Sep 21;6(9):e2334953. doi: 10.1001/jamanetworkopen.2023.34953 (PMC10514735; doi:10.1001/jamanetworkopen.2023.34953)
Supplement: Supplement 2. — Data Sharing Statement [file jamanetwopen-e2334953-s002.pdf]

Data Sharing Statement

Mefford. Incidence of Diabetes Before and During the COVID-19 Pandemic Among Youth. *JAMA Netw Open*. Published September 21, 2023. doi:10.1001/jamanetworkopen.2023.34953

Data
Data available: No

Additional Information
Explanation for why data not available: Anonymized data that support the findings of this study may be made available from the investigative team in the following conditions: 1) agreement to collaborate with the study team on all publications, 2) provision of external funding for administrative and investigator time necessary for this collaboration, 3) demonstration that the external investigative team is qualified and has documented evidence of training for human subjects protections, and 4) agreement to abide by the terms outlined in data use agreements between institutions.
